# Pseudoporphyria Associated with Nonhemodialyzed Renal Insufficiency, Successfully Treated with Oral N-Acetylcysteine

**DOI:** 10.1155/2013/271873

**Published:** 2013-04-10

**Authors:** A. C. Katoulis, D. Ferra, E. Toumbis, E. Papadavid, A. Kanelleas, I. Panayiotides, D. Rigopoulos

**Affiliations:** ^1^Second Department of Dermatology, “Attikon” General University Hospital, University of Athens, 1 Rimini Street, 124 62 Chaidari, Greece; ^2^Histopathology Department, “Attikon” General University Hospital, University of Athens, 124 62 Chaidari, Greece

## Abstract

Pseudoporphyria (PP) is a relatively rare, photodistributed bullous dermatosis that resembles porphyria cutanea tarda (PCT), but it is not accompanied by porphyrin abnormalities in the serum, urine, or stool. It was initially described in renal failure patients on dialysis. Thereafter, it has been associated with several aetiological factors. We report a case of PP in a 67-year-old woman with mild renal failure, successfully treated with N-acetylcysteine. This is the second reported case of PP developing in nondialyzed chronic renal failure. Such cases support the view that renal impairment itself may play a more important aetiological role in developing PP than it was originally considered.

## 1. Introduction

Pseudoporphyria (PP) is a relatively rare, photodistributed bullous dermatosis that clinically, histopathologically, and immunologically resembles porphyria cutanea tarda (PCT), but it is not accompanied by porphyrin abnormalities in the serum, urine, or stool. It was initially described in patients with renal failure on dialysis as “bullous dermatosis of hemodialysis” [1]. Subsequently, PP has been associated with numerous photosensitizing medications, hormone replacement, UVA radiation in tanning beds, hepatitis C, sarcoidosis, Sjogren syndrome, hepatoma, HIV infection, and lupus erythematosus. In a retrospective study of 20 cases, the mean age at diagnosis was 50 years [2]. Naproxen-induced PP and tanning-bed-induced PP most often affect women, but the latter may reflect social trends in gender use of artificial tanning. Among patients with end-stage renal disease, PP has been estimated to occur in 1.2–18% of those on hemodialysis and, less frequently, in those on peritoneal dialysis [1].

## 2. Case Presentation

**Figure 1:**
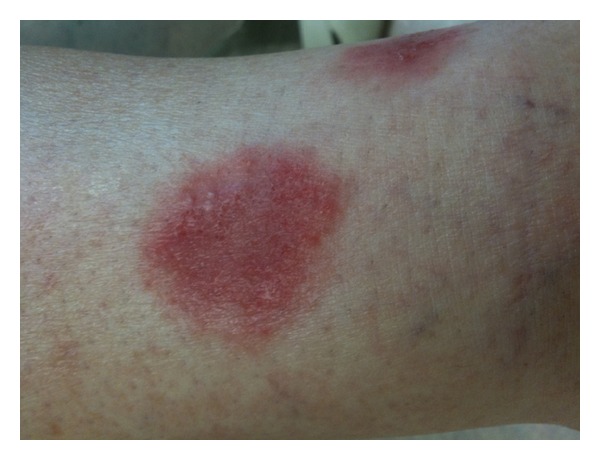
Erythematous and vesicular plaque on the shin.

**Figure 2:**
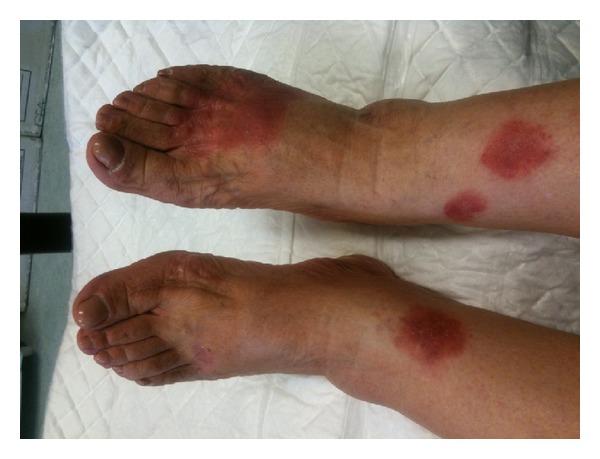
Erythematous and vesicular plaques on the shins and feet.

A 67-year-old Caucasian female presented with several erythematous lesions of the lower limbs dating 6 months previously. She had a history of hand eczema, mild chronic renal failure of unknown aetiology, hypoparathyroidism, and hypercholesterolaemia. Her family history was unremarkable, except for her mother also suffering from eczema. She was under long-term treatment with amlodipine besylate, valsartan, nebivolol hydrochloride, paricalcitol, simvastatin, allopurinol, and metformin hydrochloride. On clinical examination, several slightly pruritic, roundish, sharply demarcated, erythematovesiculosus plaques were observed, symmetrically distributed over the anterior aspect of the lower legs and feet (Figures 1 and 2). Histological examination of lesional skin showed hyperkeratosis and acanthosis of the epidermis. In the papillary dermis, we noted PAS (+), diastase (−) eosinophilic, donut-like rings around the capillaries. Direct immunofluorescence showed microgranular deposition of the C3 around the papillary dermal vessels. Porphyrin profile was within normal limits. Based on clinical and histological grounds, a diagnosis of pseudoporphyria was made. The patient was initially treated for 4 weeks with topical corticosteroids with only minimal improvement. Then N-acetylcysteine 600 mg p.o. daily for 8 weeks was given. On 1-month followup, there was a significant improvement, while in 2 months the lesions cleared completely with only residual postinflammatory hyperpigmentation. No side effects were observed.

## 3. Discussion

Clinically, PP is characterized by bullae, developing on photo-exposed skin, most commonly on the dorsum of the hands and feet, forearms, face, and neck. The lesions heal with scaring and milia formation. In contrast to PCT, hypertrichosis, hyperpigmentation, and sclerodermoid plaques only rarely occur.

Of critical importance for the diagnosis of PP is the exclusion of true porphyria, especially PCT. By definition, in PP porphyrin profile is normal or near normal [2]. Individuals with chronic renal failure tend to have higher serum porphyrin levels than normal, with some levels determined to be within the lower end of the range commonly found in PCT patients with normal renal function. Also plasma uroporphyrin levels are generally higher in patients on hemodialysis compared with those on peritoneal dialysis, which may explain the lower incidence of PP in the latter group.

The exact pathophysiological mechanism of PP is largely unknown. Formation of phototoxic metabolites in genetically predisposed individuals may trigger the development of bullous lesions. The action spectrum has generally been assumed to be in the range of ultraviolet radiation or, possibly, visible light. There is evidence that some of the causally associated drugs also induce photosensitivity. Reactive oxygen species have been incriminated in the pathogenesis of dialysis-associated PP. These patients are at high risk of oxidative stress due to deficiency of glutathione in the blood and erythrocytes, which may increase their susceptibility to the effects of UV exposure at even lower porphyrin levels. In addition, clearance of plasma-bound porphyrin precursors may lead to excessive porphyrin deposition in the skin. It could also be related to aluminum hydroxide, which is found in the dialysis solution and has been shown to produce a porphyria-like reaction to rats. 

In cases of drug-induced PP, withdrawal of the suspected photosensitizing medication results in improvement usually within weeks to months (average 8 weeks). Strict UVR protection, including a broad spectrum sunscreen, is crucial. In hemodialysis-associated PP, there are reports of complete resolution after treatment with N-acetylcysteine (800–1200 mg p.o. daily for 8 weeks), a glutathione precursor, but some authors noticed recurrence when the drug was discontinued [3–5]. Chloroquine has been tried also with satisfactory results after one month.

Our patient is the second reported case of PP developing in nondialyzed chronic renal failure. However, a drug-induced aetiology cannot be definitely excluded. The question whether it is the disease (chronic renal failure) or the management (hemodialysis) which contributes most to the occurrence of PP remains to be answered. Such cases support the view that renal impairment itself may play a more important aetiological role in developing PP than it was originally considered. Dialysis in genetically predisposed patients may indeed contribute to PP development, either by the resulting in oxidative stress or by reflecting a more advanced stage of renal failure. In addition, oral N-acetylcysteine proved to be effective in our case at a lower than the previously reported dose.
